# Nipple adenoma detected by multimodal ultrasound: a case report and literature review

**DOI:** 10.3389/fonc.2024.1457293

**Published:** 2024-10-23

**Authors:** Jianghao Lu, Jingwen Zhang, Tingting Wu, Yuqin Ma, Peng Zhou

**Affiliations:** ^1^ Department of Ultrasound, First Affiliated Hospital of Shenzhen University Health Science Center, Shenzhen Second People’s Hospital, Shenzhen, China; ^2^ Department of Pathology, First Affiliated Hospital of Shenzhen University Health Science Center, Shenzhen Second People’s Hospital, Shenzhen, China

**Keywords:** breast tumor, nipple adenoma, imaging, contrast-enhanced ultrasound, diagnosis

## Abstract

Nipple adenoma (NA) is a rare benign lesion of the lactiferous ducts, often mistaken for malignancy due to its diverse clinical and imaging presentations. We report the case of a 34-year-old female presenting with persistent bloody discharge and nipple erosion, for which multimodal ultrasound evaluation was pivotal in the differential diagnosis. Ultrasonography revealed a hypoechoic, well-defined nodule in the left nipple, with significant blood flow and a fast-in-fast-out contrast enhancement pattern, indicative of NA. Despite the presentation mimicking malignant processes, the benign nature of the lesion was confirmed postoperatively via histology and immunohistochemistry. This case underscores the value of a comprehensive ultrasound approach in diagnosing NA, emphasizing its ability to distinguish it from malignant lesions, and thus infer an appropriate treatment course. Maintaining a high index of suspicion coupled with tailored ultrasound techniques is recommended for accurate NA diagnosis, which remains a challenging yet critical task to avoid unnecessary aggressive interventions.

## Introduction

Nipple adenoma (NA) is a rare, benign, proliferative lesion of the lactiferous ducts and acini predominantly present in the periareolar region of the breast (1). Characterized by diverse clinical presentations, NA can often mimic more ominous conditions, such as Paget’s disease (PD) or invasive carcinoma, both in clinical examinations and imaging results (2). This clinical mimicry makes the definitive diagnosis of NA challenging, compounded by its rarity, which necessitates a high index of suspicion and comprehensive diagnostic work-up. Historically, NAs have been considered unusual due to their diverse presentation and broad differential diagnosis, encompassing infectious, inflammatory, and neoplastic diseases. The condition can present as a palpable nodule, nipple discharge (serous or bloody), nipple retraction, or dermatological changes resembling eczema or psoriasis, often leading to delayed diagnosis or mismanagement (3). Therefore, the significance of breast ultrasound in this context cannot be overstated. As a non-invasive, readily available, and dynamic imaging technique, ultrasonography plays a pivotal role in the initial assessment and follow-up of such lesions. It aids in delineating the size, extent, and relation of the lesion to adjacent structures and guides further diagnostic interventions (4). Despite their utility, imaging findings of NAs can be non-specific, featuring ductal dilatation, solid hypoechoic nodules, or complex cystic structures, thereby overlapping with the sonographic appearance of malignant lesions. This study aimed to discuss the complex interplay between the clinical and multimodal sonographic features of NAs, with a focus on enhancing diagnostic accuracy and optimizing management strategies.

## Case presentation

A 34-year-old woman presented to our department for ultrasonography due to persistent bloody discharge for over two years and erosion of her left nipple for more than two months. The patient had a history of cesarean section 7 years ago and resection of a benign breast tumor 2 years prior. Physical examination revealed symmetrical breasts with no localized swelling or depressions, no redness, edema, or orange peel-like changes in the skin, and no superficial vein dilation.The nipples were at the same level on both sides. The surface of the left nipple was eroded. A mobile, well-defined nodule approximately 1×1 cm in size was palpated in the nipple of the left breast. When the nipple and areola were squeezed, a light red liquid flowed out. There was no palpable mass in the right breast, and no discharge was expressed upon nipple-areolar complex palpation. No enlarged lymph nodes were palpable in the bilateral armpits, supraclavicular fossae, or subclavian fossae. The patient had no family history of breast malignancy or genetic disease.

**Figure 1:**
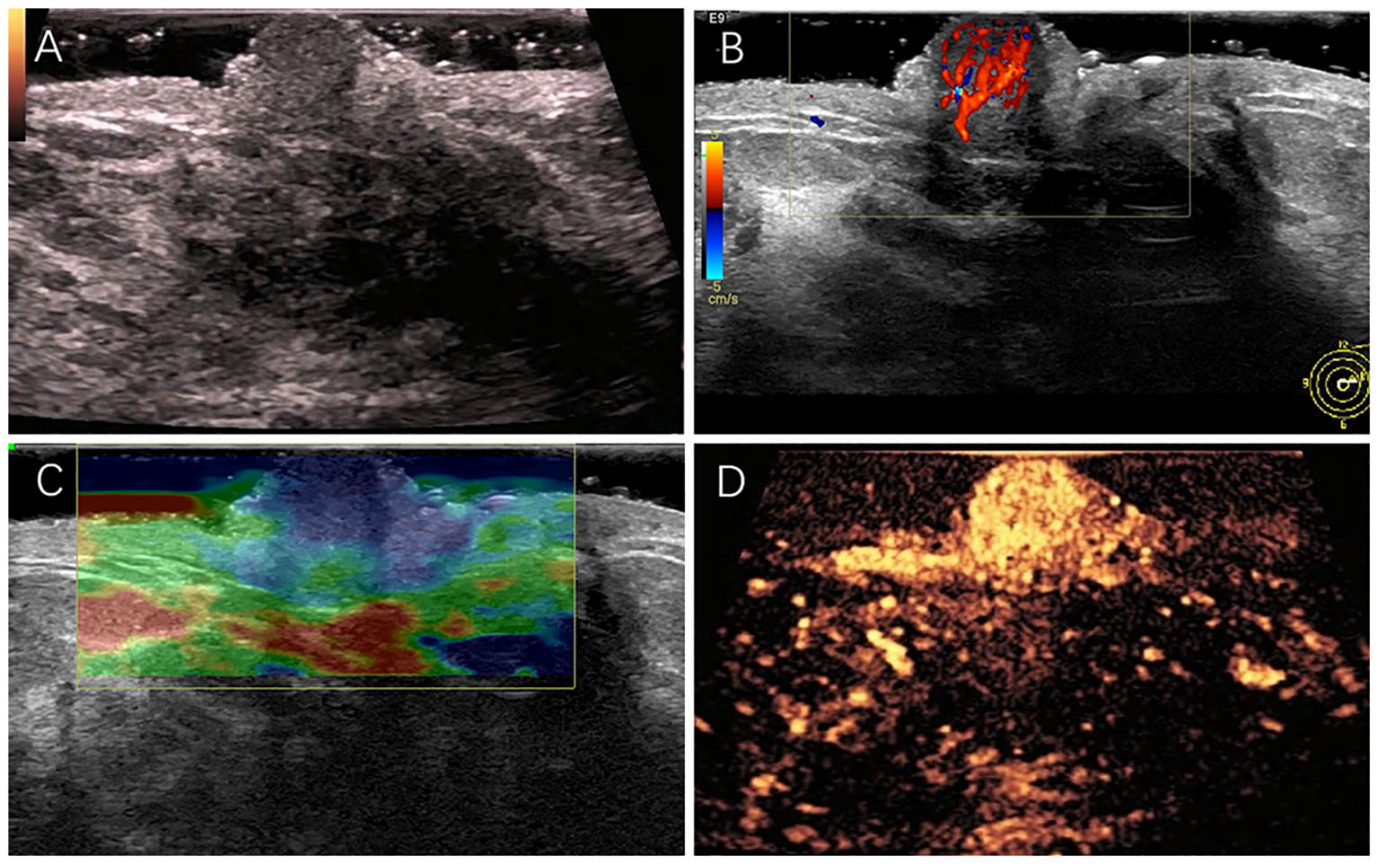
**(A)** A hypoechoic nodule measuring approximately 8×6 mm in size in the left nipple. **(B)** Rich blood flow signal detected in the nodule. **(C)** The overall texture of the nodule was harder than its surrounding tissues. **(D)** The lesion was highly enhanced with clear enhancement margin.

The patient underwent a multimodal ultrasound assessment, including gray-scale ultrasound, color Doppler flow imaging, strain elastography, and contrast-enhanced ultrasound. During contrast-enhanced ultrasound examination, we used intravenous bolus injection of sulfur hexafluoride microbubble contrast agent (SonoVue^®^, Bracco, Milan, Italy). After injecting 4.8 mL the contrast agent, enhancement of the nipple and its surrounding tissues was observed and recorded for 120s. Gray-scale ultrasonography revealed a hypoechoic lesion in the left nipple, roughly elliptical in shape, measuring approximately 8×6 mm, with clear borders and homogeneous internal echogenicity (**Figure 1A**). Color Doppler flow imaging revealed a rich blood flow signal within the lesion (**Figure 1B**). Ultrasound elastography revealed a hard texture inside the lesion (**Figure 1C**). In the CEUS examination, enhancement of breast tissues around the nipple began at 15s after injection of the contrast agent, and enhancement of the lesion in the left nipple began at 11s. The lesion reached peak enhancement at 20s and was completely washed out at 120s. Contrast-enhanced ultrasonography indicated that the lesion was heightened with clear enhancement margin, and the surrounding tissues were homogeneously enhanced. Additionally, it demonstrated earlier washout of the contrast agent in the lesion than in the surrounding tissues, indicating fast-in-fast-out enhancement, a pattern that suggests high vascularization (**Figure 1D**). Synthesizing the results of the multimodal ultrasound examinations, the lesion was finally diagnosed as ACR BI-RADS category 3, consistent with NA characteristics.

**Figure 2:**
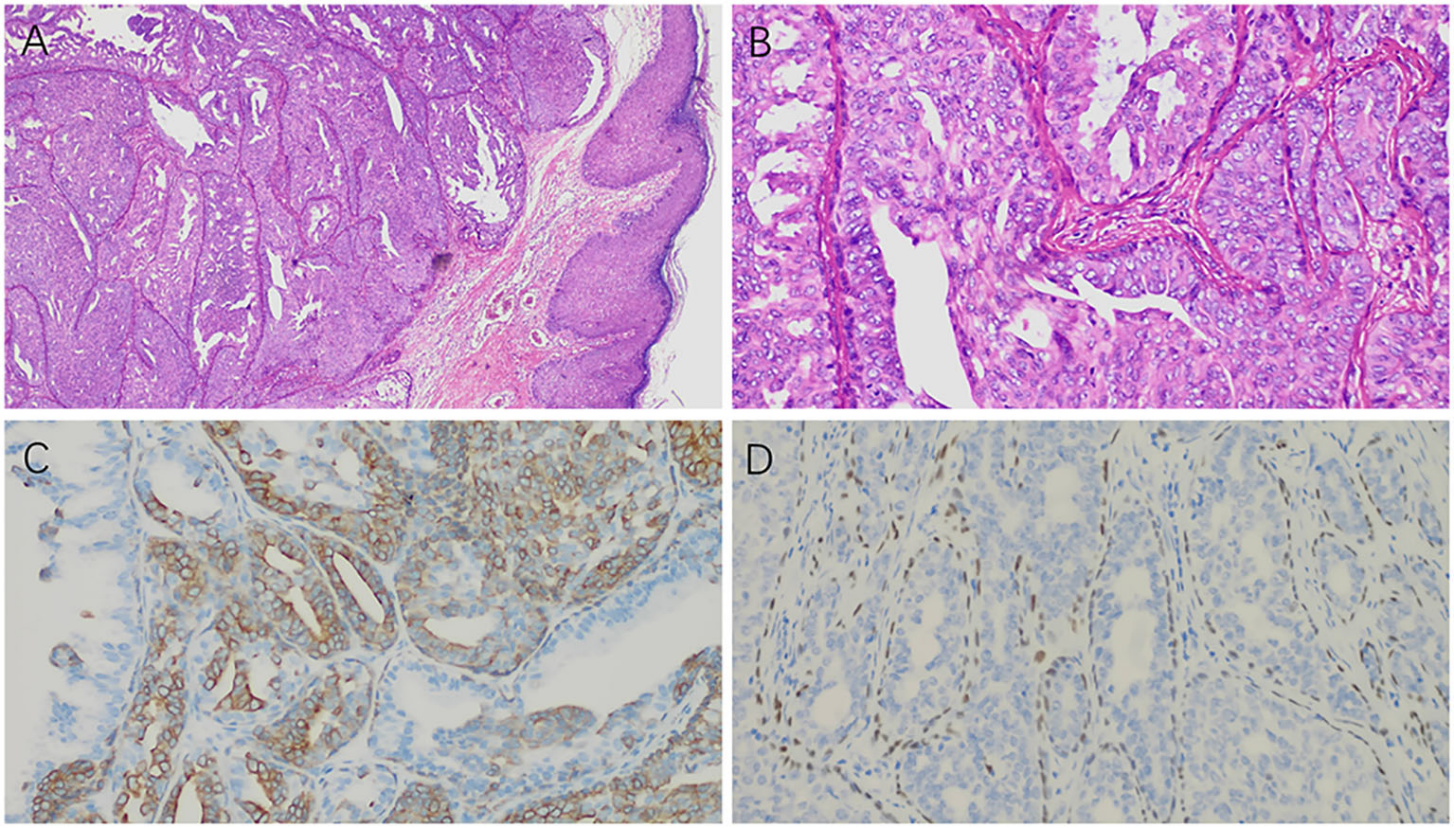
Postoperative histology and immunohistochemistry results: **(A)** Under low magnification, the ductal epithelium exhibits papillary hyperplasia (×100). **(B)** Under high magnification, the ductal epithelium shows active hyperplasia (×400). **(C)** CK5/6 staining shows positive expression in the hyperplastic ductal epithelium (×400). **(D)** p63 staining is positive in the myoepithelium surrounding the hyperplastic ductal epithelium (×400).

The patient underwent excision of the left nipple lesion under local anesthesia. The patient showed good compliance and tolerance of the intervention both during the ultrasound examination and during the operation. Postoperative histology and immunohistochemistry were consistent with a NA, characterized by hyperplasia with localized necrosis (**Figure 2**). At postoperative follow-up, the patient’s left nipple did not show symptoms similar to those before. Breast ultrasound examinations over the next three years showed no significant abnormalities. The patient expressed satisfaction with the treatment process during subsequent visits.

## Discussion

NA, also known as erosive adenomatosis, florid papillomatosis, florid adenomatosis, subareolar duct papillomatosis, and papillary adenoma, is a benign epithelial proliferative lesion of the breast characterized by duct-like structures involving the superficial duct orifices of the nipple, surrounding stroma, and often contiguous overlying epidermis (1, 5, 6). NA typically occurs in patients age 40-50s. The most common symptom is bloody nipple discharge. Pain and tenderness have rarely been reported only rarely (7). The management of NA begins with diagnostic mammography and breast ultrasonography to delineate the extent of the nipple abnormality and exclude malignancy. While mammograms are generally normal, breast ultrasonography typically shows a well-defined hypoechoic mass with internal vascularity located within the nipple (2). There are three main histological subtypes of NAs: (1) epithelial hyperplasia, with tubular, solid papillary, and pseudocribriform patterns; necrosis might be present; (2) adenosis type, where the adenoma is within the dermis; and (3) pseudoinfiltrating type, with distorted ducts and squamous cysts without epithelial hyperplasia (8). Benign proliferation of lactiferous ducts and disorganized growth of both luminal epithelial and myoepithelial cells in the ducts are important findings for distinguishing NA from invasive breast carcinoma (9). In our case, the presence of these two types of cells were confirmed using immunohistochemistry.

NAs are benign breast diseases. However, Josephine et al. reported a case of low-grade adenosquamous carcinoma arising in association with NA, indicating the possibility of progression to malignant disease (10). NAs are easily misdiagnosed as PD when ulceration occurs; however, no histological features of Paget disease are observed microscopically. These adenomas usually exhibit common ductal hyperplasia, making them difficult to differentiate from breast cancer (11). In Liu’s study, most cases of nipple PD are accompanied by malignant lesions in other areas of the breast, which can help in the differential diagnosis of NAs and PD (3). However, Sam et al. reported the case of a patient who presented with concurrent NA and breast malignancy. This patient had an adenoma in her left breast nipple and an invasive lobular carcinoma in her right breast (12). Surgical treatment and pathological evaluation are the primary approaches for definitive diagnosis to exclude potential malignancies (13, 14). However, recurrence has been reported to be as high as 25–55% following incomplete excision (15). The standard approach for the treatment of NA is complete excision of the nipple, or even the nipple-areolar complex, to ensure negative histological margins and prevent recurrence. If the tumor does not invade the dermal papilla or epidermis, Mohs microsurgery and cryotherapy may be considered to preserve the nipple integrity (16). Joshua et al. reported the case of a 26-year-old patient treated with Mohs micrographic surgery to successfully preserve the nipple-areolar complex for cosmetic purposes (17).

## Conclusion

Mammography, magnetic resonance imaging, and conventional ultrasonography have yet to be of limited benefit in the diagnosis and differentiation of NAs from malignancies. Multimodal ultrasonography including gray-scale ultrasound, color Doppler flow imaging, ultrasound elastography and contrast-enhanced ultrasound, can provide a full range of ultrasound imaging features to assist in diagnosis (18, 19). In this case, multimodal ultrasound revealed a well-defined hypoechoic nodule in the nipple with abundant blood flow, hard texture, and high enhancement in contrast mode. Moreover, the lesion exhibited well-defined margin with fast-in-fast-out enhancement and no expansion of the enhancement was observed.

## Data Availability

The original contributions presented in the study are included in the article/supplementary material, further inquiries can be directed to the corresponding author/s.

## References

[1] Lester SC Lee AHS. WHO classification of tumours authorial board. Breast tumours. In: Sasano H, editor. WHO classification of tumours of the nipple, 5th ed. IARC, Lyon, France (2019). 182–3.

[2] Rodriguez PP Crabtree M Venegas R Ozao-Choy J Dauphine C. Nipple adenoma: A benign disease with a suspicious presentation. Am surgeon. (2023) 89:6243–5. doi: 10.1177/00031348221117026 36007058

[3] Liu X Xu Y Liu J Sun S Zhu Y Lu H. Pathological and imaging features of Paget's disease and nipple adenoma: a comparative study. Gland Surg. (2022) 11:207–15. doi: 10.21037/gs-21-862 PMC8825522 35242682

[4] Chiorean A Pintican RM Szep M Feier D Rogojan L Fetica B. Nipple ultrasound: A pictorial essay. Korean J Radiol. (2020) 21:955–66. doi: 10.3348/kjr.2019.0831 PMC7369201 32677380

[5] DIB M Cantile M Collina F D'Aiuto M Liguori G DEC R. Adenoma of the nipple: A clinicopathological report of 13 cases. Oncol Lett. (2014) 7:1839–42. doi: 10.3892/ol.2014.2000 PMC4049716 24932244

[6] Dinesh BJ Hayati F Azizan N Abdul Rashid NF. Florid papillomatosis of the nipple. BMJ Case Rep. (2019) 12(9):e231516. doi: 10.1136/bcr-2019-231516 PMC6754673 31537599

[7] Weigelt MA Sciallis AP McIntire PJ Ko JS Billings SD Ronen S. Nipple adenoma: clinicopathologic characterization of 50 cases. Am J Surg Pathol. (2023) 47:926–32. doi: 10.1097/PAS.0000000000002063 37272622

[8] Spohn GP Trotter SC Tozbikian G Povoski SP. Nipple adenoma in a female patient presenting with persistent erythema of the right nipple skin: case report, review of the literature, clinical implications, and relevancy to health care providers who evaluate and treat patients with dermatologic conditions of the breast skin. BMC Dermatol. (2016) 16:4. doi: 10.1186/s12895-016-0041-6 27206635 PMC4873987

[9] Aktas E Moustafa E Uysal I Armutlugoynuk H Ozay Nayir P. Nipple adenoma in a young female: A case report. J Cosmet Dermatol. (2022) 21:5221–2. doi: 10.1111/jocd.v21.10 35034414

[10] Wilsher MJ Desai AJ Pinder SE. Low-grade adenosquamous carcinoma arising in association with a nipple adenoma. Histopathology. (2020) 76:784–7. doi: 10.1111/his.14033 31705759

[11] Fornage BD Faroux MJ Pluot M Bogomoletz W. Nipple adenoma simulating carcinoma. Misleading clinical, mammographic, sonographic, and cytologic findings. J ultrasound medicine: Off J Am Institute Ultrasound Med. (1991) 10:55–7. doi: 10.7863/jum.1991.10.1.55 1997686

[12] Alhayo ST Edirimanne S. Clinically challenging case of nipple adenoma. Breast J. (2018) 24:1084–5. doi: 10.1111/tbj.2018.24.issue-6 30033567

[13] Wang C Wang X Ma R. Diagnosis and surgical treatment of nipple adenoma. ANZ J Surg. (2015) 85:444–7. doi: 10.1111/ans.2015.85.issue-6 24975720

[14] Leo ME Carter GJ Waheed U Berg WA. Nipple adenoma: correlation of imaging findings and histopathology. J Breast Imaging. (2022) 4:408–12. doi: 10.1093/jbi/wbac019 PMC9334779 35915844

[15] Fujii T Yajima R Morita H Yamaguchi S Tsutsumi S Asao T. Adenoma of the nipple projecting out of the nipple: curative resection without excision of the nipple. World J Surg Oncol. (2014) 12:91. doi: 10.1186/1477-7819-12-91 24716784 PMC3996199

[16] Bae KN Shin K Kim WI Yang MY Lee WK Kim HS. Cryosurgery as a minimally invasive alternative treatment for a patient with erosive adenomatosis of the nipple. Ann Dermatol. (2021) 33:182–5. doi: 10.5021/ad.2021.33.2.182 PMC8081994 33935461

[17] Owen JL Krunic AL. Successful treatment of nipple adenoma using mohs micrographic surgery to preserve the nipple-areolar complex. Dermatologic surgery: Off Publ Am Soc Dermatologic Surg. (2020) 46:132–5. doi: 10.1097/DSS.0000000000001839 30789507

[18] Li M Du J Wang LJ Li Z Chen X. A case of nipple adenoma detected by sonography. Chin Med J. (2016) 129:2386–7. doi: 10.4103/0366-6999.190674 PMC5040028 27647201

[19] Zhang X Wu K Fu S. Nipple adenoma of the breast: A case report of 10-year follow-up. Asian J Surg. (2023) 46:2868–9. doi: 10.1016/j.asjsur.2023.01.078 36740522

